# Short course radiotherapy with simultaneous integrated boost for stage I-II breast cancer, early toxicities of a randomized clinical trial

**DOI:** 10.1186/1748-717X-7-80

**Published:** 2012-06-01

**Authors:** Hilde Van Parijs, Geertje Miedema, Vincent Vinh-Hung, Sylvia Verbanck, Nele Adriaenssens, Dirk Kerkhove, Truus Reynders, Daniel Schuermans, Katrien Leysen, Shane Hanon, Guy Van Camp, Walter Vincken, Guy Storme, Dirk Verellen, Mark De Ridder

**Affiliations:** 1 Department of Radiotherapy, UZ Brussel, Laarbeeklaan 101, 1090, Brussels, Belgium; 2 Department of Pneumology, UZ Brussel, Laarbeeklaan 101, 1090, Brussels, Belgium; 3 Department of Physical Therapy, UZ Brussel, Laarbeeklaan 101, 1090, Brussels, Belgium; 4 Department of Cardiology, UZ Brussel, Laarbeeklaan 101, 1090, Brussels, Belgium

**Keywords:** Early breast cancer, Hypofractionation, Simultaneous integrated boost (SIB), Image guided radiation treatment (IGRT), Intensity modulated radiotherapy (IMRT)

## Abstract

**Background:**

TomoBreast is a unicenter, non-blinded randomized trial comparing conventional radiotherapy (CR) vs. hypofractionated Tomotherapy (TT) for post-operative treatment of breast cancer. The purpose of the trial is to compare whether TT can reduce heart and pulmonary toxicity. We evaluate early toxicities.

**Methods:**

The trial started inclusion in May 2007 and reached its recruitment in August 2011. Women with stage T1-3N0M0 or T1-2N1M0 breast cancer completely resected by tumorectomy (BCS) or by mastectomy (MA) who consented to participate were randomized, according to a prescribed computer-generated randomization schedule, between control arm of CR 25x2 Gy/5 weeks by tangential fields on breast/chest wall, plus supraclavicular-axillary field if node-positive, and sequential boost 8x2 Gy/2 weeks if BCS (cumulative dose 66 Gy/7 weeks), versus experimental TT arm of 15x2.8 Gy/3 weeks, including nodal areas if node-positive and simultaneous integrated boost of 0.6 Gy if BCS (cumulative dose 51 Gy/3 weeks). Outcomes evaluated were the pulmonary and heart function. Comparison of proportions used one-sided Fisher's exact test.

**Results:**

By May 2010, 70 patients were randomized and had more than 1 year of follow-up. Out of 69 evaluable cases, 32 were assigned to CR (21 BCS, 11 MA), 37 to TT (20 BCS, 17 MA). Skin toxicity of grade ≥1 at 2 years was 60% in CR, vs. 30% in TT arm. Heart function showed no significant difference for left ventricular ejection fraction at 2 years, CR 4.8% vs. TT 4.6%. Pulmonary function tests at 2 years showed grade ≥1 decline of FEV1 in 21% of CR, vs. 15% of TT and decline of DLco in 29% of CR, vs. 7% of TT (P = 0.05).

**Conclusions:**

There were no unexpected severe toxicities. Short course radiotherapy of the breast with simultaneous integrated boost over 3 weeks proved feasible without excess toxicities. Pulmonary tests showed a slight trend in favor of Tomotherapy, which will need confirmation with longer follow-up of patients.

**Trail registration:**

ClinicalTrials.gov NCT00459628

## Background

Breast cancer is the most frequently diagnosed cancer and the leading cause of cancer death in women, accounting worldwide for 23% of total new cancer cases and 14% of total cancer deaths in 2008 [1]. The past decades have seen advances in the diagnosis and treatment of breast cancer, associated with a decrease of mortality rate, although the changes vary widely between countries [2,3]. Among treatments, adjuvant radiotherapy has shown to improve local control and overall survival, with a 70% proportional reduction of the risk of recurrence [4] and a 9%–12% proportional reduction of the risk of death [5-8]. Despite this established role of radiotherapy, there are considerable disparities in the receipt of radiotherapy that are attributable to various factors such as limited availability of treatment centers, geographical distance, long waiting times, and costs [9-11]. The disparities can further be compounded by the long schedules required with conventional radiotherapy, since the schedules of radiotherapy that were evaluated in clinical trials and were found to be associated with improved survival are based on conventional fractionation of 1.8-2.5 Gy/fraction, delivering treatment over 5 to 7 weeks [5,8,12,13]. Many researches are actively investigating alternative approaches. Intraoperative radiotherapy (IORT) [14,15] or accelerated partial breast irradiation (APBI) [16] provide the shortest schedules. However, IORT and APBI are limited to selected cases of breast conservation therapy [17]. Whole breast radiotherapy with a hypofractionated schedule delivering 42.5 Gy in 16 fractions over 22 days has been shown by the Ontario randomized trial to be comparable with a conventional schedule of 50 Gy in 25 fractions over 35 days [18]. However, boost radiation was not used. The UK START trial A and trial B found that 41.6 Gy in 13 fractions over 5 weeks or 40 Gy in 15 fractions over 3 weeks given after breast conserving surgery (BCS) or after mastectomy (MA) had outcomes on local control and adverse effects comparable to the conventional treatment of 50 Gy in 25 fractions over 5 weeks [19,20]. A boost of 10 Gy in 5 fractions was allowed to centers that elected to give boost, as well as regional radiotherapy to supraclavicular nodes with or without axillary chains. The issue of boost radiation was not addressed in the Ontario trial, for this reason boost was given by conventional fractionation in the UK START trials, reducing the gain in scheduling time.

In the present study, we designed an experimental hypofractionated schedule that would shorten overall treatment time and be applicable to mastectomy patients as well as to breast conservation patients by integrating a simultaneous boost. The experimental treatment is compared with conventional radiotherapy in a randomized clinical trial (NCT00459628). The present study reports early toxicities.

## Methods

The primary outcome measure defined in the trial was the change from baseline in pulmonary and heart function tests up to 3 years after treatment. The secondary outcome measures were local-regional recurrences. The trial started recruiting patients in May 2007. Eligible patients were women aged 18 years or older, presenting with histologically proven breast carcinoma, operated by BCS or MA with clear margins, pathological stage T1-3N0M0 or T1-2N1M0 [21]. Availability of at least one pre-operative imaging by CT, MRI, and/or PET-scan was required. Exclusion criteria were history of prior breast or thoracic radiotherapy, pregnancy or lactation, absence of effective contraception in fertile patients, psychiatric or addictive disorders.

After written informed consent, patients were randomized to either a control arm of conventional radiotherapy (CR), or to the experimental arm of hypofractionated Tomotherapy (TT). Randomization was balanced by nodal status, type of surgery and chemotherapy sequence using Efron's biased coin design [22].

In the control arm, a dose of 50 Gy was delivered in 25 fractions over 5 weeks to the chest wall (MA) or the whole breast (BCS) by 6 or 15 MV photons tangential wedged fields and using field-in-field multileaf compensation when doses exceed 110%, and to the supraclavicular, infraclavicular and axillary nodes in case of pN1 status, using an anterior 6 MV photons half-beam matched to the superior border of the tangential fields. The field borders were set clinically. The typical tangential field borders were: superior just below the clavicle head, inferior 1.5 cm below the infra-mammary crease or the lower part of the ipsilateral breast (BCS) or the contralateral breast (MA), medial at mid-sternum and lateral at the mid-axillary line (pN0) or at the anterior border of the scalene muscles (pN1). The borders of the supraclavicular field were: superior caudal to the cricoid cartilage, inferior at the caudal edge of the clavicle head, medial excluding the trachea and lateral at the junction of the first rib with the clavicle. Breast conserved patients received an additional boost of 16 Gy in 8 fractions over 2 weeks to the initial tumor bed using a direct electron field, i.e. a cumulative dose of 66 Gy in 33 fractions over 7 weeks at the tumor bed. No dose constraints for lung and heart were defined in the conventional arm, but the perpendicular distance from the chest wall to the posterior field edge preferably included no more than 2 cm of lung at any point along the length of the tangent. This lung distance was not to exceed 3 cm. For left-sided breast radiotherapy, the maximum heart distance was not to exceed 1.5 cm. The boost volume was aligned taking into account the pre-operative imaging (mammography and CT, MRI or PET) and post-operative changes (scar, seroma) seen on the planning-CT. A clinical target volume (CTV) -margin of 7 mm was used. Often surgical clips at the borders of the operation area were available. These clips were to be inside the CTV. CTV to planning target volume (PTV) -margin was 5 mm.

In the experimental arm, patients were treated using the Helical TomoTherapy® Hi-art system [23] (Madison, US). A total dose of 42 Gy in 15 fractions over 3 weeks was prescribed to the same target volumes as the conventional arm: chest wall in case of MA or whole breast in case of BCS, and to the supraclavicular, infraclavicular and axillary nodes in case of pN1 status. In case of BCS, a simultaneous integrated boost (SIB) of 0.6 Gy per fraction was prescribed to the tumor bed, i.e. a dose of 51 Gy in 15 fractions over 3 weeks at the tumor bed. The boost volume was aligned as for the conventional radiotherapy, with the same CTV and PTV margins. Tomotherapy dose specifications for target volumes breast/chest wall, boost, and lymph node regions were to receive 95%–105% of prescribed dose. Dose constraints for heart and ipsilateral lung were respectively V5Gy < 10% and V17Gy < 7%, contralateral breast V10Gy < 5%.

Per protocol, radiotherapy in any arm started within 6 weeks after breast surgery. In case of sequential adjuvant treatment with chemotherapy first, radiotherapy started after a delay of 2 weeks, but within 6 weeks after completion of adjuvant chemotherapy. Concomitant chemotherapy was allowed.

Pulmonary and heart function, arm mobility and arm lymphedema were assessed prior to radiotherapy, at 2 (± 1) months after completion of radiotherapy and thereafter yearly for 3 years. Heart function was assessed by measuring the left ventricular ejection fraction (LVEF) by echocardiography. Pulmonary function tests assessed forced expiratory volume in one second (FEV1) and diffusing capacity of the lung for carbon monoxide (DLco). Heart and lung toxicity scoring was based on the common terminology criteria of adverse events (CTCAE) v.3 [24]. Scoring of breast/chest wall skin and subcutaneous toxicity used the Radiation Therapy Oncology Group (RTOG) acute (up to 1 month post radiotherapy) and late (after 1 month) morbidity scoring schemas [25]. Scoring of arm lymphedema used the SOMA/LENT toxicity scale [26].

The planned accrual was to recruit 118 patients, based on a power of 0.80 to detect a 5% vs. 25% incidence of all heart and lung toxicities between the two treatment arms. The trial closed for inclusion in August 2011. A total of 123 patients were randomized after giving informed consent. The present report concerns the first 70 patients enrolled up to August 2009, having a minimal follow-up time of 1 year. The follow-up cut-off date was May 2011. Data were analyzed by intention to treat. Comparison of proportions used Fisher's exact test, one-sided. Statistical computations used JMP v. 8.0.1 (SAS Institute Inc, Cary, NC, USA). The trial was accepted by the ethical committee of the UZ Brussel.

## Results

Of the 70 women with more than 1 year follow-up, 1 patient was not eligible due to bilateral breast carcinoma, leaving 69 patients available for the present study. Thirty two were randomized to CR, 37 to TT. Two patients allocated to CR refused conventional treatment and were subsequently treated by TT. One patient allocated to TT could not be positioned on the Tomotherapy couch because of extreme obesity, she was treated by CR.

The mean age of the study participants was 55 years (range 32--78 years) (Table1). There was a non-significant preponderance of left sided tumors, 58% (40 of 69). A medial tumor location was observed in 29% (20 of 69) patients. Breast conserving surgery was performed in 59% (41 of 69) patients. Axillary surgery was by sentinel nodes biopsy alone in 54% (37 of 69), sentinel nodes biopsy followed by completion axillary lymph node dissection in 14% (10 of 69), and axillary lymph node dissection by first intent in 32% (22 of 69). The pathological mean tumor size was 17 mm (range 2--42 mm). Multifocality was noted in 14% (10 of 69) patients. The majority of patients were node negative, 65% (45 of 69). The mean lymph node ratio (LNR) among node-positive patients was 0.13 (range 0.04--0.33). Using cut-offs of 0.20 and 0.65 [27], 79% (19 of 24) of the node-positive patients had a low LNR, 21% (5 of 24) had an intermediary LNR, and no patient had a high LNR. The frequencies of estrogen receptor positivity, progesterone receptor positivity, and HER2 FISH amplification were 80% (55 of 69), 67% (46 of 69), and 14% (10 of 69), respectively. A high histological grade was observed in 26% (18 of 69) patients. Adjuvant chemotherapy was given to 49% (34 of 69) patients. Among patients receiving adjuvant chemotherapy, most had chemotherapy scheduled to start concomitantly with radiotherapy, 76% (26 of 34), i.e. 38% of all patients. Adjuvant hormone therapy was given to 80% (55 of 69) and trastuzumab to 14% (10 of 69) patients.

**Table 1:** Patients’ characteristics

	**Control**	**Tomotherapy**
	**n = 32**	**n = 37**
Age		
<50	10	15
> = 50	22	22
Left breast tumor	16	24
Medial location	12	8
Tumor size		
T1 (<= 20 mm)	21	26
T2 (21–50 mm)	11	11
Nodal status, Lymph node ratio		
pN0	21	24
pN1, LNR 0.01-0.20	7	12
pN1, LNR 0.21-0.65	4	1
pN1, LNR > 0.65	0	0
Estrogen receptor positive	28	27
Progesterone receptor positive	21	25
HER2 FISH amplified	2	8
Histological grade		
1	11	11
2	8	18
3	10	8
unknown	3	0
Surgery		
Breast conserving	21	20
Axillary lymph nodes		
Sentinel biopsy only	20	17
Sentinel with axillary dissection	4	6
Immediate axillary dissection	8	14
Radio-chemotherapy schedule		
No adjuvant chemotherapy	18	17
RT after completion of chemotherapy	3	5
RT concomitant with start chemotherapy	11	15
Chemotherapy type		
Anthracycline without taxane	3	4
Anthracycline with taxane	10	15
CMF	1	1
Hormone therapy		
No hormone therapy	4	10
Tamoxifen	15	11
Letrozole	13	11
Zoladex	0	1
Tamoxifen + Zoladex	0	4
Trastuzumab	2	8

The distribution of the patients' characteristics by age, laterality, tumor location, type of surgery, histopathology, hormone receptor status, tumor size, lymph node positivity and ratio, adjuvant chemotherapy and hormone therapy were comparable between the two randomization arms (Table1).

**Figure 1:**
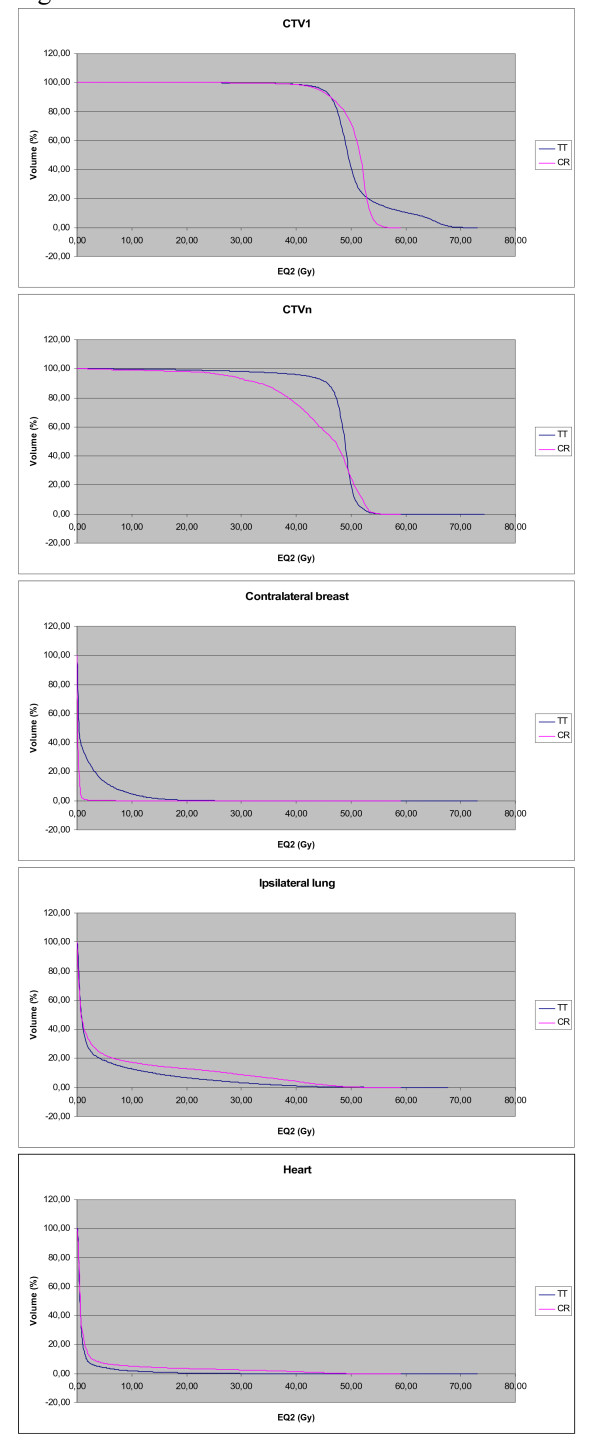
**Dose volume histogram.** The average dose volume histograms (DVH) of the CTV's and the most important organs at risk are shown for CR and TT. The average DVH for the heart was generated for left sided irradiation only. The dose is formulated in 2 Gy equivalent dose (EQ2) with an alpha/beta of 3 Gy.

Figure1 shows the average dose volume histograms (DVH) for the breast/chest wall (CTV1), the lymph node areas (CTVn), the contralateral breast, the ipsilateral lung for all patients and the heart for left sided irradiation. This shows a more homogeneous coverage of the CTV1 and the CTVn in the Tomotherapy group. We compare the CTV and not the PTV data, because no PTV is made for the conventional treatment, since the borders of the irradiation field for this group are chosen according to clinical landmarks. A small margin is allowed in the Tomotherapy group, since daily megavolt CT imaging is performed before every treatment session. Notice the tail in the CTV1 of the Tomotherapy group. This is explained by the dose gradient from the CTV1 to the boost volume (CTVb). This is not present in the conventional therapy, because the boost in this group is given after the end of the whole breast irradiation. This additional boost is given with electrons. Our planning system does not allow a dose calculation for electrons. This means that the DVHs for the conventional therapy lack part of the actual received dose. Even so there is fewer dose to the heart and ipsilateral lung with Tomotherapy. The average dose on the heart is 7.1 Gy ± 5.7 Gy (CR), 1.7 Gy ± 2.5 Gy (TT), on the ipsilateral lung is 6.6 Gy ± 12.0 Gy(CR) and 4.7 Gy ± 7.5 Gy (TT). A higher dose is delivered on the contralateral breast with Tomotherapy compared to the conventional treatment (average dose CR: 0.3 Gy ± 0.5 Gy vs. TT: 2.6 Gy ± 2.3 Gy). These findings are in line with the results of a planning study we performed earlier [23].

The median follow-up of all patients was 28 months (range 16--48 months). No patient died during follow-up. There was no local recurrence or new primary breast tumor. One patient in the control arm was diagnosed with bone metastases 2 months after randomization. A second primary tumor was diagnosed in 3 patients in the control arm (1 skin basal cell carcinoma of the nose, 1 kidney carcinoma, 1 sigmoid carcinoma) and in 1 patient in the Tomotherapy arm (1 lung adenocarcinoma) (P = 0.3).

All patients completed radiotherapy. Mean treatment duration was 43 days (range 20--54) in the conventional arm, 22 days (range 18--36) in the Tomotherapy arm. At completion and up to 1 month after radiotherapy, the clinical evaluation and RTOG acute scoring found skin toxicity grade 0 in 6.25% (2 of 32), grade 1 in 65.6% (21 of 32), grade 2 in 21.9% (7 of 32), and grade 3 in 6.25% (2 of 32) of patients in the control arm; grade 0 in 5.4% (2 of 37), grade 1 in 59.5% (22 of 37), grade 2 in 27.0% (10 of 37), grade 3 in 8.1% (3 of 37) in the Tomotherapy arm (P Chi-square = 0.94). Peak erythema occurred at the end of treatment in the conventional arm and at 10 to 14 days after end of treatment in the Tomotherapy arm.

**Figure 2:**
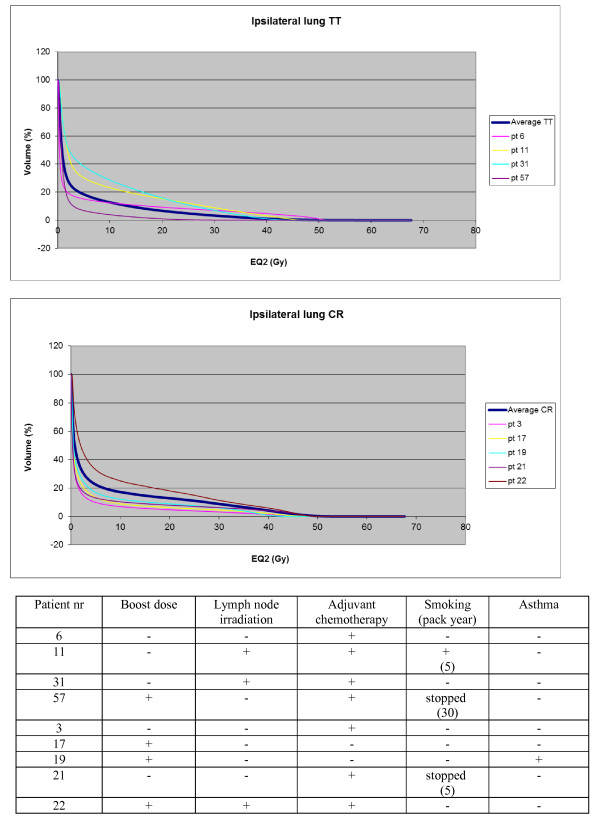
**DVHs of the ipsilateral lung for patients who showed consistent DLco-decrease of >10%.** The individual DVHs for the patients who had G1 or higher lung toxicity based on DLco, which proved consistent over time, are shown compared to the average DVH for TT and CR. The dose is formulated in 2 Gy equivalent dose (EQ2) with an alpha/beta of 3 Gy.

Table2 summarizes toxicities observed from 2 months up to 2 years. We pooled all toxicities of grade 1 or higher as compared with pre-radiotherapy baseline. At 2 years, patients in CR had twice more persistent skin change than patients in the TT, 60% (12 of 20 patients) vs. 30% (6 of 20 patients), P = 0.06. The frequencies of breast/chest wall fibrosis, arm lymphedema and heart toxicity (LVEF) were comparable between the two randomization arms. Lung function tests showed a significant reduction of lung toxicity in TT based on changes of DLco, P = 0.047, but not based on changes of FEV1. Figure2 shows the individual DVHs for the patients who had G1 or higher lung toxicity based on DLco, which showed consistent over time, compared to the average DVH. The individual DVHs are positioned above, as well as below the average DVH. In the situation that the observed lung toxicity would be closely related to the delivered lung dose, one would expect all the individual DVHs above the average DVH. Among these 9 women 7 were treated with concomitant chemotherapy, of which 3 were irradiated on the lymph node regions. A boost dose was delivered in 4 women. Three women had a smoking history. One woman had known asthma.

**Table 2:** Occurrence of toxicity grade ≥1 change from baseline after radiation treatment, by intent to treat randomization arm

	**Control**			**Tomotherapy**			**P-value 1 sided**
	**Number of patients evaluated**	**Observed grade ≥1**	**% grade ≥1**	**Number of patients evaluated**	**Observed grade ≥1**	**% grade ≥1**	
**Skin**							
at 2 months	32		65.6	21	36	24	66.7	0.636
at 1 year	31	14	45.2	36	14	38.9	0.393	
at 2 years	20	12	60.0	20	6	30.0	*0.056*	
**Breast/chest wall fibrosis**								
at 1 year	32	12	37.5	36	10	27.8	0.276	
at 2 years	24	9	37.5	24	6	25.0	0.267	
**Arm lymphedema**								
at 2 months	32	2	6.2	36	1	2.8	0.455	
at 1 year	32	2	6.2	36	2	5.6	0.647	
at 2 years	24	3	12.5	24	3	12.5	0.667	
**Heart function**								
at 2 months	32	9	28.1	35	4	11.4	0.078	
at 1 year	31	4	12.9	36	8	22.2	0.906	
at 2 years	21	1	4.8	22	1	4.6	0.744	
Lung toxicity score based on change in FEV1 (*)								
at 2 months	30	4	13.3	34	3	8.8	0.429	
at 1 year	29	4	13.8	34	4	11.8	0.552	
at 2 years	24	5	20.8	27	4	14.8	0.422	
Lung toxicity score based on change in DLco (*)								
at 2 months	30	10	33.3	34	8	23.5	0.277	
at 1 year	29	8	27.6	34	3	8.8	0.052	
at 2 years	24	7	29.2	27	2	7.4	*0.047*	
Lung toxicity based on change in FEV1 and DLco (*)								
at 2 months	30	12	40.0	34	10	34.3	0.266	
at 1 year	29	12	41.4	34	6	28.6	0.036	
at 2 years	24	10	41.7	27	5	22.2	*0.066*	

## Discussion

A meta-analysis performed in our department, using data of the Early Breast Cancer Trialists' Cooperative Group (EBCTCG), argued that trials showing a survival benefit were the more recent trials using current techniques [5]. Considering that the survival advantage with adjuvant radiotherapy was confirmed [6,28], the question that arises is whether or not breast cancer could further benefit from more advanced radiotherapy techniques. There are however two obstacles against the implementation of image guided radiotherapy (IGRT) for breast cancer patients. One obstacle is that IGRT is labor intensive. Priority to implement IGRT is given to the treatment of tumor conditions in which adverse reactions can be severe and debilitating, such as lung cancer, digestive or head and neck tumors [29,30]. The other obstacle is the controversy on whether IGRT could improve over simpler variants of tangential fields [31]. There is thus a need to prospectively assess the benefit of IGRT for breast cancer patients.

The earlier mentioned meta-analysis also argued that a survival benefit was shown in trials using a standard fractionation between 1.8 Gy and 2.5 Gy per fraction [5]. Since then, there has been growing evidence on the applicability of hypofractionation for adjuvant radiotherapy of breast cancer, as shown in Table3. A natural extension is therefore to combine hypofractionation with IGRT in order to improve the availability of IGRT to breast cancer patients. Our clinical trial is the first to compare an integrated strategy of hypofractionated IGRT, versus a strategy of conventional radiotherapy.

**Table 3:** Randomized clinical trials of hypofractionated whole breast/chest wall radiotherapy

**Trial**	**Period**	**n**	**Hypofraction schedule**	**SIB**	**Mastectomy**	**Regional nodes**	**IMRT/ IGRT**	**Chemo-therapy**	**Outcome**
Hôpital Necker (*) [32]	1982-	230	5.75 Gy x 4 F/ 17	No	Yes	?	NS	Yes	=
	1984		days						
Royal Marsden Hospital [33]	1986-	1410	3 Gy x 13 F/ 5	No	No	Yes	NS	No	More local relapses
	1998		weeks						
Royal Marsden Hospital			3.3 Gy x 13 F/ 5	No	No	Yes	NS	No	=
			weeks						
Ontario [18]	1993-	1234	2.66 Gy x 16 F/ 3	No	No	No	NS	Yes	=
	1996		weeks						
UK Start A [19]	1998-	2236	3 Gy x 13 F/ 5	No	Yes	Yes	NS	Yes	More local relapses
	2002		weeks						
UK Start A			3.2 Gy x 13 F/ 5	No	Yes	Yes	NS	Yes	=
			weeks						
Lahore [34]	1998-	300	5.4 Gy x 5 F/ 1	No	Yes	Yes	NS	Yes	=
	2004		week						
Lahore			3.5 Gy x 10 F/ 2	No	Yes	Yes	NS	Yes	=
			weeks						
Lahore			2.66 Gy x 15 F/ 3	No	Yes	Yes	NS	Yes	Control arm
			weeks						
UK Start B [20]	1999-	2215	2.67 Gy x 15 F/ 3	No	Yes	Yes	NS	Yes	=
	2001		weeks						
Egypt NCI [35]	2002-	30	2.66 Gy x 16 F/ 3	No	No	No	NS	No	Boost in conventional arm
	2003		weeks						
UK FAST [36]	2004-	915	5.7 Gy x 5 F/ 5	?	No	No	Yes	No	=
	2007		weeks						
UK FAST			6 Gy x 5 F/ 5	?	No	No	Yes	No	More breast toxicity
			weeks						
DBCG 2009 RT Hypo [37]	2009-	1500**	2.67 Gy x 15 F/ 3	No	No	No	Yes	Yes	On-going
			weeks						
UK IMPORT HIGH	2009-	840**	2.4 Gy [SIB 3.2 Gy]	Yes	No	?	Yes	?	On-going
			x 15 F/						
UK IMPORT HIGH			2.4 Gy [SIB 3.53 Gy]	Yes	No	?	Yes	?	
			x 15 F/						
UZ Brussel	2007-	122	2.8 Gy [SIB 3.4 Gy]	Yes	Yes	Yes	Yes	Yes	Closed for inclusion; FU on-going
[this study]			x 15 F/ 3 weeks						

(*) Partial report; full study 1982–1989, n = 525 patients. (**) Planned accrual.

NS: not stated. =: outcome comparable with control arm. SIB: simultaneous integrated boost. FU: follow-up.

Our choice of fractionation of 42 Gy to whole breast and boost to 51 Gy in 15 fractions took into account that our institution's standard conventional radiotherapy is 50 Gy to whole breast and boost to 66 Gy. In the IMPACT HIGH trial, the schedule in 15 fractions is to deliver 36 Gy to whole breast, and simultaneous integrated boost to 48 Gy - 53 Gy, which based on an alpha/beta of 3 Gy would correspond to conventional schedule of 40 Gy whole breast and boost to 60 Gy - 69 Gy [38].

Table3 shows how our study compares with other clinical trials. We are aware of several controversial issues, notably regarding post-mastectomy radiotherapy for node-negative patients and for node-positive patients with less than 4 nodes involved. We have argued that these patients did derive a survival benefit [39,40] and have maintained the treatment of nodal areas in node-positive patients regardless of the number of involved nodes. Most recently, results from the MA 20 trial confirmed that regional nodal irradiation prolonged disease free survival for women with one to three involved nodes [41].

Another controversial issue is the high number of patients treated by concomitant chemo-radiotherapy. One randomized trial found a significantly better loco regional recurrence free survival with a concomitant approach in node-positive breast cancer, with an acceptable increase in toxicity [42,43]. More data with current chemotherapy regimens, including taxanes, will need to be accrued.

There is the issue of delivering a simultaneous integrated boost, giving an even higher daily dose to the tumor bed. We could show this did not deliver a higher dose to the organs at risk. This technique could prove advantageous by even delivering less total dose to the surrounding breast tissue [44], though this was not analyzed in detail. Longer follow up is needed to compare the long term side effects.

The trial compares two treatment strategies. Differences that arise between the treatment arms cannot be unequivocally attributed to fractionation or to technique. A 2x2 design would have been conceptually more satisfying, but was practically unfeasible.

Regarding the trial's primary endpoint, the results showed a trend of reduced lung toxicities but not significant enough to warrant early stopping of the trial. No excess toxicities were found in the Tomotherapy arm, which argues that hypofractionation with simultaneous integrated boost is feasible, when using IMRT-IGRT. This can have important implication regarding availability and accessibility of advanced radiotherapy techniques to more patients. Hypofractionation not only is more convenient for the patients by limiting the number of treatment attendances, it can reduce waiting times for adjuvant radiation therapy and possibly impact on survival [45]. Shorter schedules can have an important socio-economic impact by lowering treatment costs due to reduced resource use in terms of personnel and machine time [46]. An increase in costs can be expected due to the more labor intensive preparation of a Tomotherapy treatment and a longer daily machine time for treatment delivery and related quality assurance. But the most important determinant of the cost of radiotherapy is the total number of fractions. A favorable balance can be expected for the hypofractionated schedule.

## Conclusions

The present analysis shows that hypofractionation with simultaneous integrated boost is feasible, when using IMRT-IGRT, without excess toxicities. There is a trend of reduced lung toxicity in the hypofractionated arm.

## Abbreviations

APBI, Accelerated partial breast irradiation; BCS, Breast conserving surgery; CR, Conventional radiotherapy; CTCAE, Common terminology criteria of adverse events; CTV, Clinical target volume; DLco, Diffusing capacity of the lung for carbon monoxide; DVH, Dose volume histogram; EBCTCG, Early Breast Cancer Trialists' Cooperative Group; EQ2, 2 Gyequivalent dose; FEV1, Forced expiratory volume in one second; IGRT, Image guided radiation treatment; IMRT, Intensity modulated radiotherapy; IORT, Intraoperative radiotherapy; LNR, Lymph node ratio; LVEF, Left ventricular ejection fraction; MA, Mastectomy; PTV, Planning target volume; RTOG, Radiation Therapy Oncology Group; SIB, Simultaneous integrated boost; TT, Tomotherapy.

## Competing interest

The department had a research agreement with TomoTherapy Inc., Madison, WI.

## Authors’ contributions

HVP drafted the manuscript, participated in the coordination of the study and had a substantial contribution to the acquisition of data. GM helped to draft the manuscript and has made a contribution to the acquisition of data. VVH conceived of the study, participated in its design and coordination, performed the statistical analysis, has made a contribution to the acquisition of data and helped to draft the manuscript. SV performed the technical analysis of the lung function tests and helped to draft the manuscript. NA performed the physical assessment and helped to draft the manuscript. DK performed the technical analysis of the heart function tests. TR and KL performed the treatment planning and planning analysis and helped to draft the manuscript. DS participated in the coordination of the study, performed the lung function tests and helped to perform the technical analysis. SH participated in the design of the study and its coordination and helped to draft the manuscript. GVC, WV and GS participated in the design of the study and revised the manuscript. DV has revised the manuscript. MDR has helped in drafting the manuscript and revised it. All authors read and approved the final manuscript.
